# Supplementary material for: Systematic Review and Patient‐Level Meta‐Analysis of SARS‐CoV‐2 Viral Dynamics to Model Response to Antiviral Therapies
Source: Clin Pharmacol Ther. 2021 May 1;110(2):321–33. doi: 10.1002/cpt.2223 (PMC8014833; doi:10.1002/cpt.2223)
Supplement: Supplementary file 1 — Supplementary Material [file CPT-110-321-s001.docx]

**Supplementary Material for “Systematic review and patient-level meta-analysis of SARS-CoV-2 viral dynamics to model response to antiviral therapies”**

**Supplementary Tables**

**Table S1** Extracted viral load data metrics

| Sample site | *n* samples (%<LOD) | Viral load metrics [log10 copies/mL] |  |
| --- | --- | --- | --- |
|  |  | Median (IQR) | [min – max] |
| Blood | 268 (79.9) | 3.96 (1.65) | 2.03 – 7.11 |
| Saliva/Throat | 2144 (37.1) | 4.62 (2.23) | 1.02 – 10.78 |
| Lower respiratory | 799 (28.9) | 5.47 (2.71) | 1.08 – 10.82 |
| Stool/rectal | 655 (35.4) | 5.05 (2.25) | -0.25 -11.38 |
| Urine | 138 (88.4) | 4.5 (1.22) | 3.76 – 6.35 |
| Breast milk | 90 (45.6) | 3.95 (2.63) | 1.96 – 5.31 |
| Ocular secretion | 50 (84.0) | 5.6 (1.99) | 2.85 -8.74 |
| Nasal | 2208 (20.8) | 5.00 (2.57) | 1.4 – 11.5 |

**Table S2** Summary of observed drug therapies – mono and combination therapies.

| Drugs | | IDs | Samples |
| --- | --- | --- | --- |
|  | No drug | 67 | 862 |
| Monotherapies |  |  |  |
|  | Azithromycin | 1 | 1 |
|  | Chloroquine/ hydroxychloroquine | 15 | 96 |
|  | Interferon | 14 | 41 |
|  | Lopinavir/ritonavir | 84 | 598 |
|  | Remdesivir | 6 | 31 |
|  | Ribavirin | 2 | 6 |
|  | Umifenovir | 16 | 68 |
|  | Thymalfasin | - | - |
| Combination Therapies |  |  |  |
|  | Azithromycin + chloroquine/ hydroxychloroquine | 6 | 40 |
|  | Azithromycin + lopinavir/ritonavir | 5 | 29 |
|  | Azithromycin + lopinavir/ritonavir + ribavirin | 5 | 35 |
|  | Azithromycin + interferon + lopinavir/ritonavir + ribavirin | 2 | 11 |
|  | Hydroxychloroquine + interferon + thymalfasin | 1 | 1 |
|  | Hydroxychloroquine + lopinavir/ritonavir | 1 | 20 |
|  | Interferon + thymalfasin | 5 | 16 |
|  | Interferon + lopinavir/ritonavir | 2 | 8 |
|  | Interferon + ribavirin | 1 | 1 |
|  | Interferon + ribavirin + umifenovir | 1 | 1 |
|  | Interferon + umifenovir + thymalfasin | 3 | 4 |
|  | Lopinavir/ritonavir + ribavirin | 33 | 222 |
|  | Lopinavir/ritonavir + umifenovir | 1 | 1 |
|  | Lopinavir/ritonavir + interferon + ribavirin | 54 | 352 |
|  | Ribavirin + umifenovir | 1 | 2 |
|  | Umifenovir + thymalfasin | 1 | 2 |

**Table S3** Parameter estimates (relative standard errors, %RSE) from NLME model fit to available sample sites.$\beta$, rate target cells become infected; $\delta$, death rate of infected cells; $\gamma$, maximum rate of viral replication; $V_{0}$, the initial viral load. Urine and ocular $V_{0}$ were not estimable and fixed to LLOD/2 with $\gamma$ fixed to e^1^

|  | Blood | Saliva/ Throat | Lower respiratory | Stool/ rectal | Urine | Breast milk | Ocular secretion | Nasal |
| --- | --- | --- | --- | --- | --- | --- | --- | --- |
| Typical estimates |  |  |  |  |  |  |  |  |
| $\boldsymbol{\beta}$ [copies x day/ml] | 0.0238 (29) | 7.25 x 10^-4^ (6) | 6.14 x 10^-6^ (6.5) | 1.37 x 10^-5^ (24) | 0.160 (15) | 2.07 (1838) | 4.70 x 10^-3^ (16) | 3.04 x 10^-5^ (19) |
| $\boldsymbol{\delta}$ [1/day] | 1.40 (230) | 0.588 (21) | 0.638 (46) | 0.572 (101) | 1.46 (110) | 1.56 (142) | 1.77 (30) | 0.796 (80) |
| $\boldsymbol{V}_{\mathbf{0}}$ [copies/ml] | 1.84 (415) | 6.62 x 10^4^ (3) | 2.42 x 10^6^ (3) | 1.91 x 10^4^ (7) | 46.06* | 237.46 (358) | 42.52* | 66 x 10^4^ (2) |
| $\boldsymbol{\gamma}$ [1/day] | 1.76 (51) | 2.46 (11) | 6.36 (31) | 1.16 (38) | 2.72* | 1.27 (342) | 2.72* | 2.13 (257) |
| Variance estimate %CV (%RSE) |  |  |  |  |  |  |  |  |
| $\boldsymbol{\beta}$ | 232 (88) | 246 (44) | 439 (37) | 332 (35) | 67.7 | 415 (720) | 149 (394) | 352 (163) |
| $\boldsymbol{\delta}$ | 62.9 (122) | 87.5 (19) | 110 (35) | 87.0 (45) | 51.5 | 564 (884) | 47.2 (219) | 86.4 (49) |
| $\boldsymbol{V}_{\mathbf{0}}$ | 483 (51) | 383 (12) | 282 (28) | 494 (29) | 570 | 510 (839) | 809 (195) | 443 (13) |
| $\boldsymbol{\gamma}$ | 10* | 10* | 10* | 0* | 10* | 10* | 10* | 10* |
|  |  |  |  |  |  |  |  |  |
| Residual error | 35 | 9.26 | 9.2 | 3.76 | 76.6 | 3.84 | 56 | 3.16 |
| Residual error, nested | 1.69 | 2.37 | 1.01 | 5.15 |  | 4.11 |  | 3.99 |

* fixed Parameters

**Table S4** Parameter estimates (relative standard errors, %RSE) from NLME model including covariate relationships. $\beta$, rate target cells become infected; $\delta$, death rate of infected cells; $\gamma$, maximum rate of viral replication; $V_{0}$, the initial viral load. Exponents of log-normal distributed parameters are reported. Drug effects can be interpreted as proportional change in δ.

|  | Parameter estimates (RSE%) | Bootstrap 95% CI |
| --- | --- | --- |
| Typical estimates |  |  |
| $\boldsymbol{\beta}$ [copies x day/ml] | 1.02 x 10^-3^ (11) | 4.69x10^-6^ – 0.078 |
| $\boldsymbol{\delta}$ [1/day] | 0.60 (10) | 0.43 – 1.93 |
| $\boldsymbol{V}_{\mathbf{0}}$ [copies/ml] | 5.97 x 10^5^ (2) | 3.83 x 10^5^ – 1.14 x 10^6^ |
| $\boldsymbol{\gamma}$ [1/day] | 2.29 (47) | 2.10 – 2.62 |
| LRT on $\boldsymbol{\beta}$ ^++^ | 9.4 x 10^-4^ (0.03) | 2.34 x 10^-8^ – 0.025 |
| IFN on $\boldsymbol{\delta}$ ^#^ | 1.75 (0.06) | 1.05 – 2.66 |
| LPVR/r on $\boldsymbol{\delta}$ ^#^ | 1.30 (0.07) | 0.75 – 1.86 |
| Riba on $\boldsymbol{\delta}$ ^#^ | 1.47 (0.02) | 1.04 – 2.47 |
| Age on $\boldsymbol{\delta}$ ^+^ | -0.07 (0.01) | -0.21 - -0.03 |
| Sex on $\boldsymbol{\delta}$ ^++^ | -0.15 (0.01) | -0.64 – 0.02 |
| Variance estimate | **%CV (RSE%) [shrinkage]** |  |
| $\boldsymbol{\beta}$ | 437 (10) [41] | 231 – 568 |
| $\boldsymbol{\delta}$ | 60.2 (6) [24] | 42.0– 75.5 |
| $\boldsymbol{V}_{\mathbf{0}}$ | 373 (6) [11] | 294 – 408 |
| $\boldsymbol{\gamma}$ | 10* |  |
|  |  |  |
| Residual error | 1.16 (95) [6] | 0.02 – 18.9 |
| Residual error, nested | 7.95 (15) [6/2/0/2] | 5.09 – 10.12 |

* fixed Parameter
Covariate effects: # proportional effect, + power function, ++ absolute change

**Table S5** Some example sample sizes (number of patients per arm drug versus placebo) required to detect a significant difference in the proportion of undetectable viral loads after 7 days of treatment with the antivirals selected by the NLME model. Power was set at 90% for a 2-sided test and significance level of p<0.05.

| Drug | Start Day 1 | Start Day 3 | Start Day 7 |
| --- | --- | --- | --- |
| Ribavirin | 185 | 225 | 317 |
| Interferon | 77 | 81 | 127 |
| Interferon+ribavirin | 23 | 27 | 41 |

**Supplementary Figures**

**
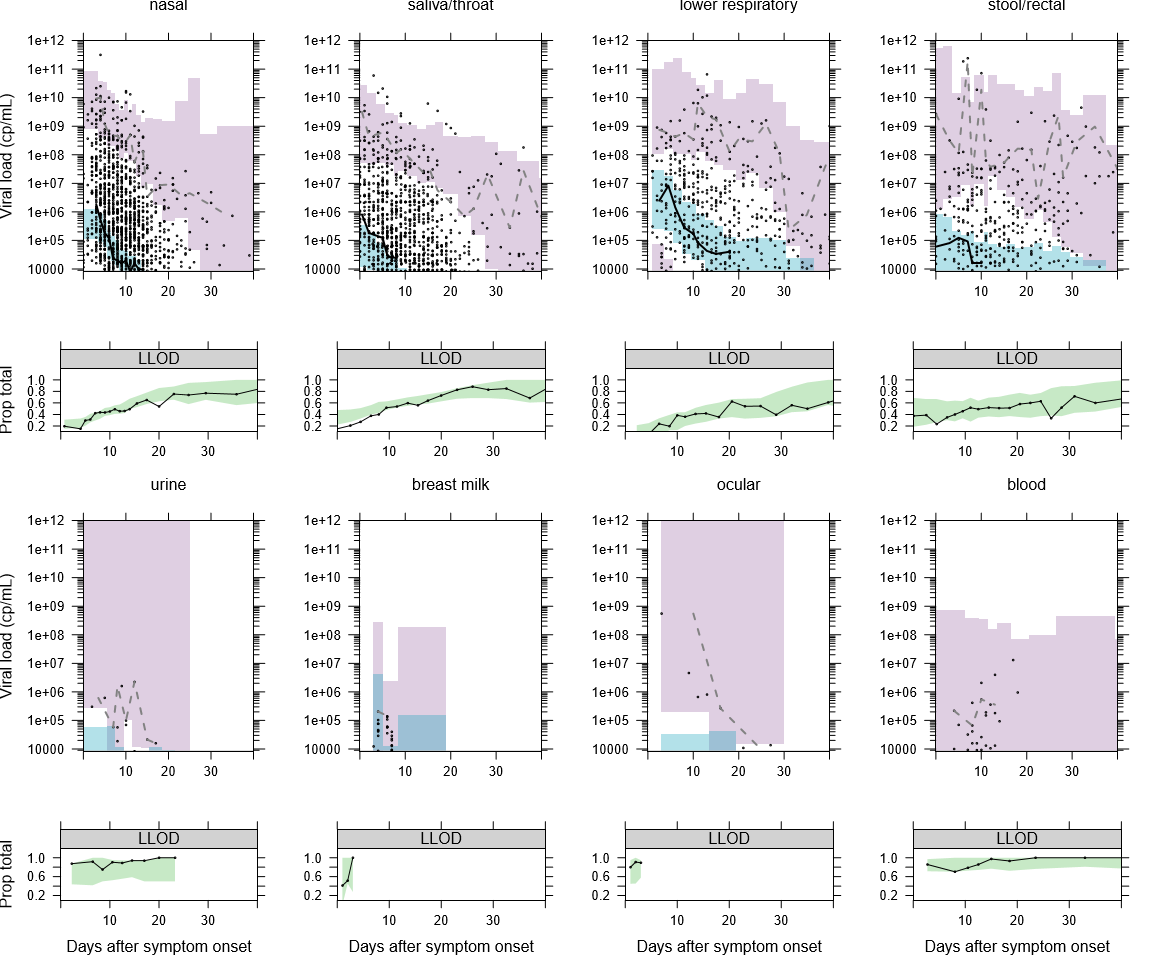
**

**Figure S1 Visual predictive checks for the NLME model fitted to viral load data to each sampling site**. For each site a plot of model simulations compared with observations is given for both the continuous data (upper) and the fraction of samples below the limit of detection (lower). Black circles are observed viral loads, purple shaded area is the 95% prediction interval of the simulated 2.5^th^ and 97.5^th^ percentile for comparison with the observed 2.5 and 97.5^th^ percentile (dashed lines). The blue shaded area is the 95% prediction interval of the 50^th^ percentile to compare with the continuous black line. In the lower plot the observed proportion of samples below the lower limit of detection (LLOD) are shown as a black line and compared with the 95% prediction interval of the model predicted proportion of samples below the LLOD (green shaded area)

**
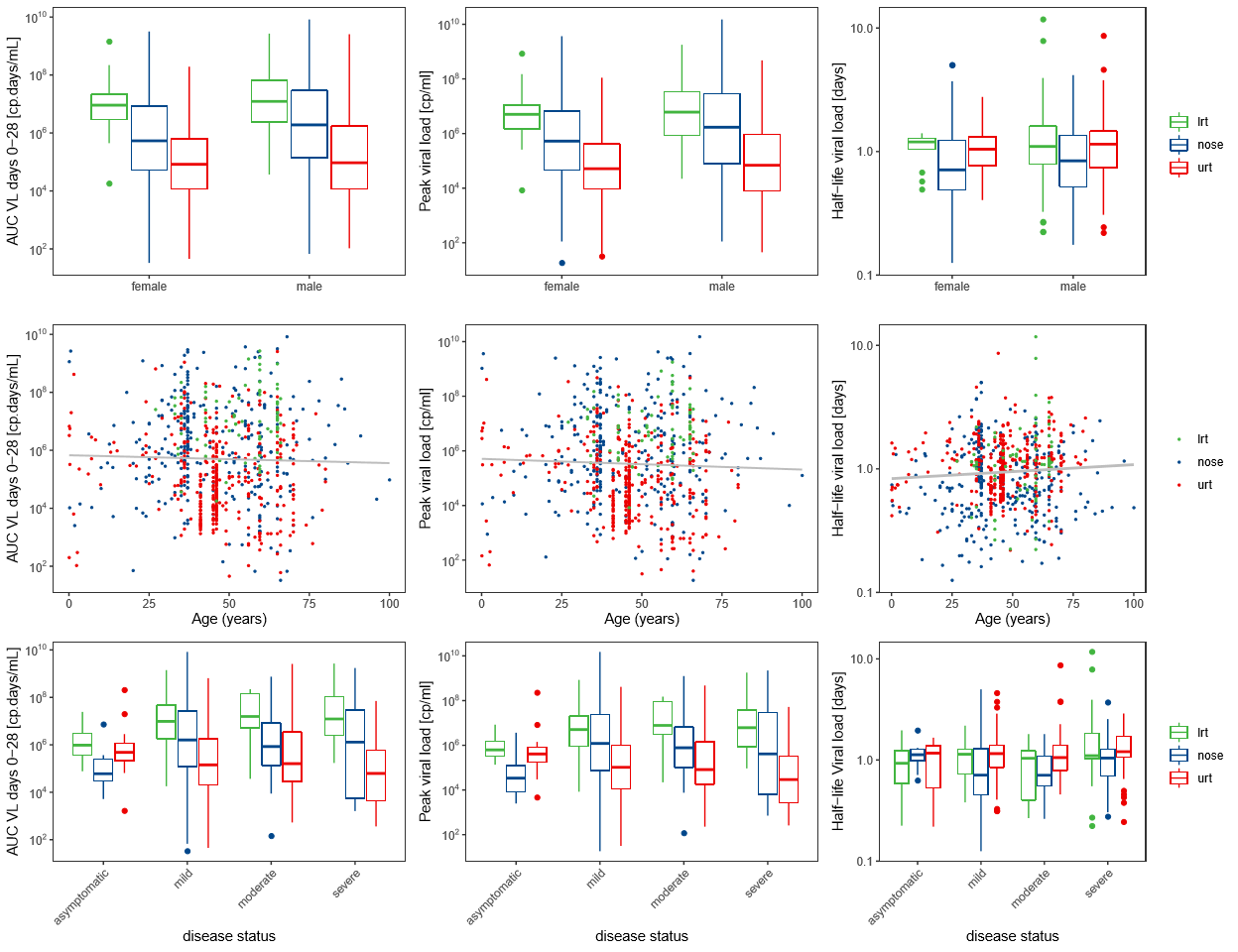
**

**Figure S2 NLME model derived parameters for respiratory sample sites.** Plot of model-predicted viral area under the curve from day 0-28 of symptom AUC_(0-28)_, peak viral load and viral elimination half-life compared with sex, age and disease severity. lrt, lower respiratory tract; urt, upper respiratory tract

**
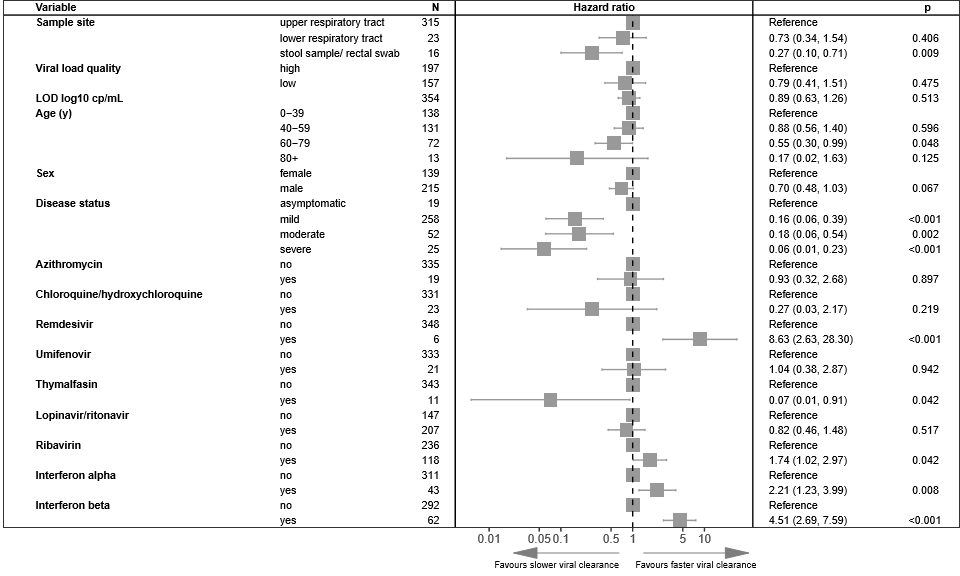
**

**Figure S3 Multivariable Cox proportional hazard results on all drug quality 1 and 2 data from respiratory and stool/rectal sampling sites with interferons split between alpha and beta.** Adjusted hazard ratios exceeding 1 indicate virus being more likely to become undetectable


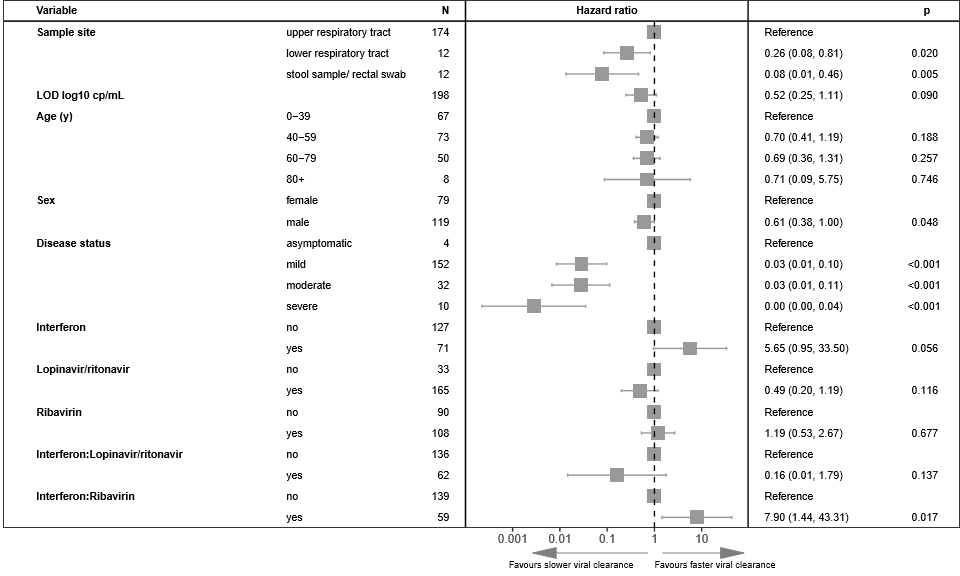


**Figure S4 Multivariable Cox proportional hazard results on viral load quality 1 data from respiratory sampling sites and stool/rectal sampling sites.** Adjusted hazard ratios exceeding 1 indicate virus being more likely to become undetectable


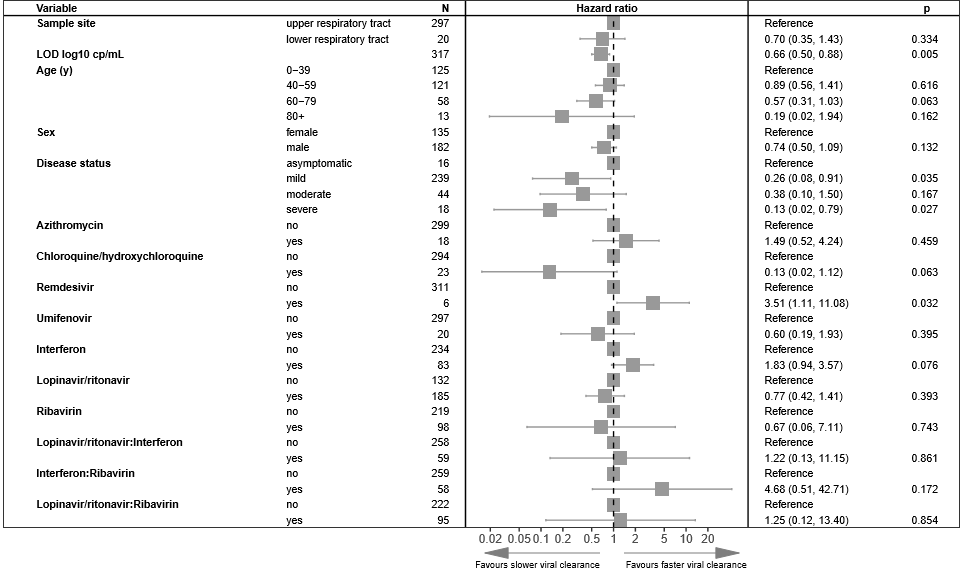


**Figure S5 Multivariable Cox proportional hazard results on viral load quality 1 data from respiratory sampling sites only.** Adjusted hazard ratios exceeding 1 indicate virus being more likely to become undetectable


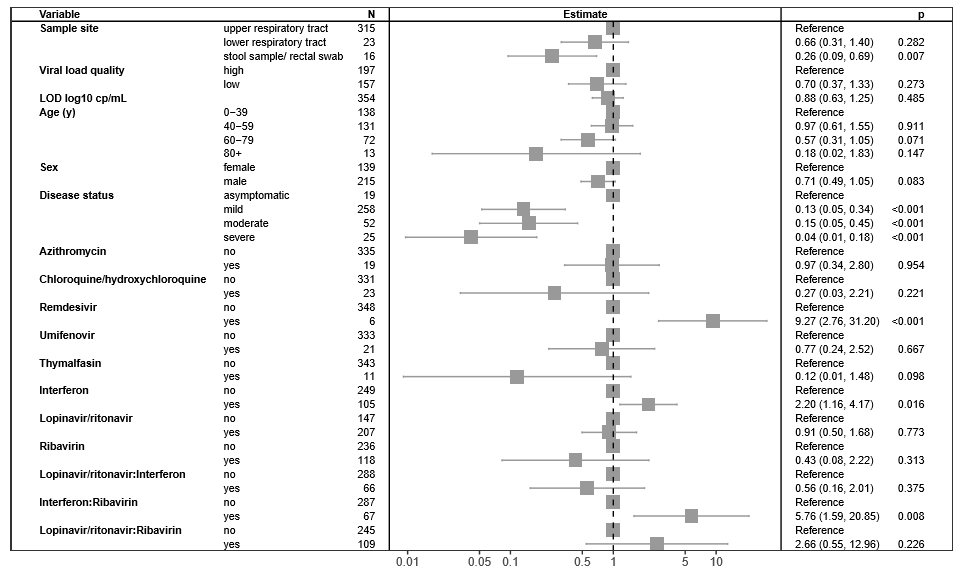


**Figure S6 Multivariable Cox proportional hazard results data excluding events with sampling frequencies over 3 days**. Adjusted hazard ratios exceeding 1 indicate virus being more likely to become undetectable


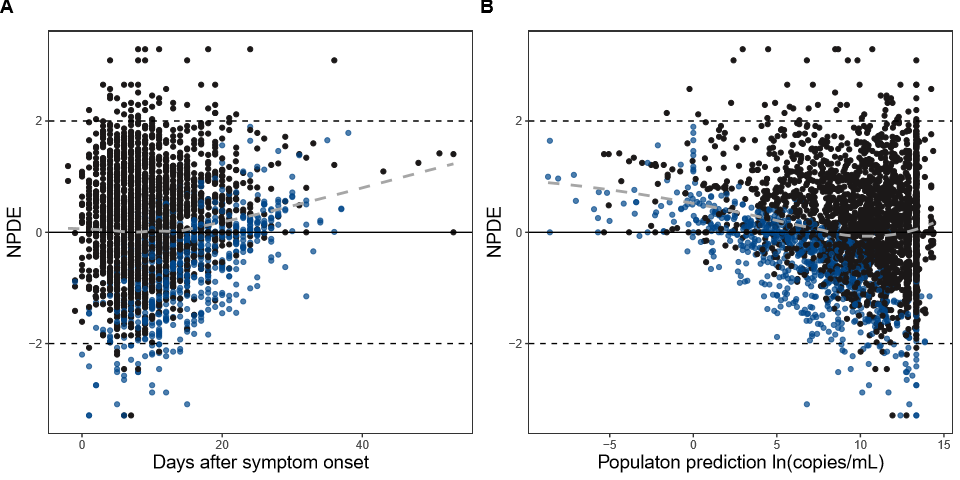


**Figure S7 Numerical prediction distribution errors (NPDE) based diagnostics for final model.** Black dots, data above lower limit of detection; blue dots, data below limit if detection


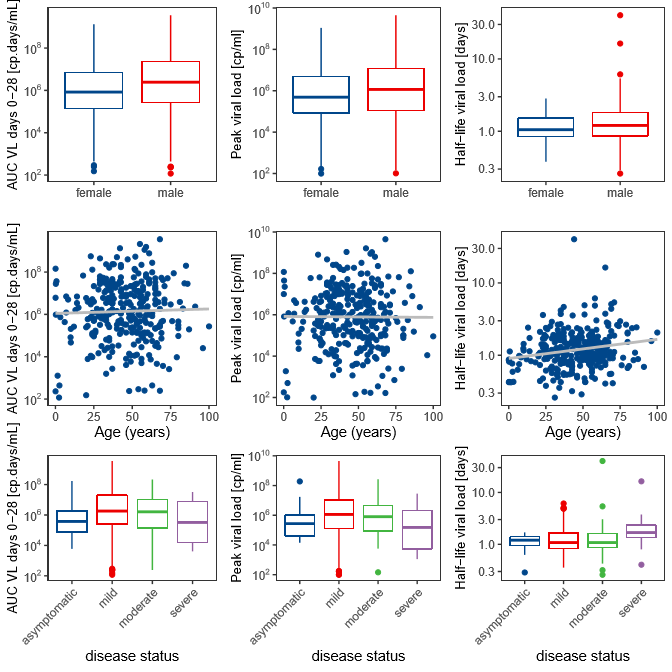


**Figure S8 NLME model derived parameters versus covariates for final model.** Plot of model-predicted viral area under the curve from day 0-28 of symptom onset; area under the viral load curve AUC(0-28), peak viral load and viral elimination half-life compared with sex, age and disease severity
